# Artificial Intelligence in the Evaluation and Intervention of Developmental Coordination Disorder: A Scoping Review of Methods, Clinical Purposes, and Future Directions

**DOI:** 10.3390/children13020161

**Published:** 2026-01-23

**Authors:** Pantelis Pergantis, Konstantinos Georgiou, Nikolaos Bardis, Charalabos Skianis, Athanasios Drigas

**Affiliations:** 1 Net Media Lab & Mind & Brain R&D, Institute of Informatics & Telecommunications, National Centre of Scientific Research ‘Demokritos’, 153 41 Athens, Greece; 2 Department of Information & Communication Systems Engineering, University of the Aegean, 832 00 Samos, Greece; cskianis@aegean.gr; 3 Occupational Therapy Department, School of Health Sciences, University of Western Macedonia, 502 00 Ptolemaida, Greece; kgeorgiou@uowm.gr; 4 Mathematics and Engineering Science Sector, Hellenic Army Academy, 166 73 Vari, Greece; nbardis@sse.gr

**Keywords:** developmental coordination disorder, dyspraxia artificial intelligence, machine learning, motor assessment, rehabilitation

## Abstract

**Highlights:**

**What are the main findings?**

- AI applications in DCD research are mainly focused on screening and assessment, with very limited attention being given to intervention.
- Most studies rely on supervised machine learning, while advanced approaches such as multimodal systems or generative AI are essentially absent.

**What are the implications of the main findings?**

- AI currently supports early identification and motor assessment in DCD but is not yet widely used to enhance therapeutic intervention.
- Future research should prioritize clinically integrated, OT- and PT-centered AI tools to support personalized intervention and functional outcomes to populations with DCD.

**Abstract:**

**Background:** Developmental coordination Disorder (DCD) is a prevalent and persistent neurodevelopmental condition characterized by motor learning difficulties that significantly affect daily functioning and participation. Despite growing interest in artificial intelligence (AI) applications within healthcare, the extent and nature of AI use in the evaluation and intervention of DCD remain unclear. **Objective:** This scoping review aimed to systematically map the existing literature on the use of AI and AI-assisted approaches in the evaluation, screening, monitoring, and intervention of DCD, and to identify current trends, methodological characteristics, and gaps in the evidence base. **Methods:** A scoping review was conducted in accordance with the PRISMA extension for Scoping Reviews (PRISMA-ScR) guidelines and was registered on the Open Science Framework. Systematic searches were performed in Scopus, PubMed, Web of Science, and IEEE Xplore, supplemented by snowballing. Peer-reviewed studies applying AI methods to DCD-relevant populations were included. Data was extracted and charted to summarize study designs, populations, AI methods, data modalities, clinical purposes, outcomes, and reported limitations. **Results:** Seven studies published between 2021 and 2025 met the inclusion criteria following a literature search covering the period from January 2010 to 2025. One study listed as 2026 was included based on its early access online publication in 2025. Most studies focused on AI applications for assessment, screening, and classification, using supervised machine learning or deep learning models applied to movement-based data, wearable sensors, video recordings, neurophysiological signals, or electronic health records. Only one randomized controlled trial evaluated an AI-assisted intervention. The evidence base was dominated by early-phase development and validation studies, with limited external validation, heterogeneous diagnostic definitions, and scarce intervention-focused research. **Conclusions:** Current AI research in DCD is primarily centered on evaluation and early identification, with comparatively limited evidence supporting AI-assisted intervention or rehabilitation. While existing findings suggest that AI has the potential to enhance objectivity and sensitivity in DCD assessment, significant gaps remain in clinical translation, intervention development, and implementation. Future research should prioritize theory-informed, clinician-centered AI applications, including adaptive intervention systems and decision-support tools, to better support occupational therapy and physiotherapy practice in DCD care.

## 1. Introduction

DCD is a neurodevelopmental condition that is prevalent, highly co-occurring, persistent, and varies in severity [1,2,3]. According to [2,4,5], the individual’s primary challenges are related to motor learning, resulting in a range of deficiencies in gross and fine motor skills that adversely affect their autonomy and functionality in their daily activities.

The relationship between DCD and neurological basis was examined by numerous scholars. Researchers discovered numerous correlations with fundamental brain regions and structures, including the mesencephalon, basal ganglia, cerebellum, parietal lobe, and thalamus, as well as their connections, using a variety of neuroimaging techniques [6,7,8,9,10]. A number of skill issues have been identified based on fine motor skills, writing, construction, obstacle avoidance, dynamic and static balance, divided attention, ball skills, response time, and movement time [11]. These issues are related to the functions of the frontal cortex as well as the synthesis and maturation of the white and gray matter of the brain.

Further knowledge includes more variable kinematics and kinetics, practice- and context-dependent motor learning, voluntary gaze control, cognitive-motor integration, and internal modeling [7,12,13]. According to [14,15], all of these symptoms may contribute to secondary issues related to social relationships, low self-esteem, depressive illnesses, and emotional/behavioral disorders. ASD, particularly learning difficulties, and ADHD are among the most frequent comorbid disorders, with DCD being the most common. These conditions share many executive function-related traits [16,17]. Research indicates that ADHD is the neurodevelopmental disorder that most frequently and significantly correlates with DCD, and that people with DCD frequently struggle with inhibition and impulsivity [18]. In order to identify and address issues that arise in the growth and maturity of the brain, the scientific community has concentrated its efforts on determining the source of these two events. There are notable differences in how the two populations present the motor deficiency from both a neurophysiological and a neurofunctional perspective, according to recent research that used neuroimaging techniques to study them [19,20].

According to several studies, DCD impairs a person’s overall functioning and causes a multitude of disturbances in the abilities and tasks that a person typically develops. The majority of them discuss motor learning challenges associated with issues such as balance, gait control, motor coordination, and visual motor impairments. Due to the intensity of their symptoms, many children with DCD also struggle to participate in school and successfully integrate into the school environment. The pooled prevalence for handwriting and math difficulties was 84.4% and 89.5%, respectively, according to the 24-study systematic review by [21], which aimed to compile all the information regarding the prevalence and extension of these difficulties among DCD children. However, no pooled prevalence for other difficulties could be ascertained. More precisely, it was said that all children with DCD performed poorly in the areas of reading, math, writing, and handwriting legibility.

To summarize, the main issues observed in it are associated with challenges in the person’s executive and motor learning abilities. Researchers are attempting to determine the cause of DCD by primarily using neuroimaging techniques, examining deviations from normally developing brains and distinctions from other neurodevelopmental disorders (such as ASD and ADHD), even though the concept has been consolidated in the DSM since 1994 [22]. Results are inconsistent and not generalizable, due to the high comorbidity with other disorders, particularly ADHD, and the unmet exclusion requirements for the research processes that are conducted.

Shifting to the impact of Industry 4.0 and the development of contemporary technology, modern science is working to achieve digitalization and develop a virtual framework that integrates the coexistence of digital, physical, and biological systems [23]. Artificial Intelligence (AI) and the development of intelligent machine systems that can analyze complicated data and problems make AI one of the most important technologies of the future. Its integration into human civilization and the ongoing, progressive developments in its field necessitate flexibility to adjust to new information and improve our daily life. Not because of the concept of “artificial,” but rather because of the question of what might be deemed to be “intelligence,” definitions for AI as a qualifier for a technological artifact may be more difficult than definitions of the field [24]. In this way, these definitions frequently speak of obstacles that must be overcome for an artifact to be deemed intelligent. Finding a minimum set of qualities that a system must possess in order to be classified as an AI is another definitional strategy. According to the AI Act of the European Union, an AI system is “a machine-based system designed to operate with varying levels of autonomy, that may exhibit adaptiveness after deployment and that, for explicit or implicit objectives, infers, from the input it receives, how to generate outputs such as predictions, content, recommendations, or decisions that can influence physical or virtual environments” [25]. “To perform, task solve, communicate, interact, and act logically as it occurs with biological humans” is another way to define AI as a qualification [26,27]. In order to construct AI-based models, according to [28], AI methods may be categorized into ten groups: (1) machine learning; (2) deep learning and neural networks; (3) data mining, knowledge discovery, and advanced analytics; (4) rule-based modeling and decision making; (5) fuzzy logic-based approach; (6) expert system modeling, knowledge representation, and uncertainty reasoning; (7) case-based reasoning; (8) text mining and natural language processing; (9) visual analytics, computer vision, and pattern recognition; (10) hybridization, searching, and optimization. Depending on the nature of the issue and the intended solution, these methods can be crucial in creating intelligent and smart systems in a variety of real-world application domains, such as business, finance, healthcare, agriculture, smart cities, cybersecurity, and many more.

The purpose of this scoping review is to systematically map and synthesize the existing literature on the use of AI in the evaluation and intervention of DCD. Specifically, this review aims to identify how AI-based and AI-assisted approaches have been applied across different stages of the DCD care pathway, including screening, assessment, monitoring, and intervention, and to describe the types of AI methods, data modalities, study designs, and target motor domains represented in the current evidence base. In addition, this scoping review seeks to characterize the populations and demographic profiles included in AI-related DCD research, examine the methodological maturity and clinical positioning of existing studies, and summarize the reported outcomes and limitations of AI applications in this field. By doing so, the review aims to highlight current research trends, gaps, and future directions, thereby informing researchers, clinicians, and developers about the potential and challenges of integrating AI into the assessment and management of DCD. Given the emerging and heterogeneous nature of AI research in DCD, a scoping review approach was selected to provide a comprehensive overview of the field rather than to evaluate intervention effectiveness or comparative efficacy of interventions.

## 2. Materials and Methods

### 2.1. Study Design

The PRISMA statement for Scoping Reviews (PRISMA-ScR) was used in this study design to apply a systematic mapping approach of the literature. The process of identifying, selecting, evaluating, and synthesizing the included studies ensured the following methodological steps were carried out. The scoping review methodology was registered using the Open Science Framework accessed on 25 December 2025 (https://osf.io/q42ns).

The scoping review design was deemed appropriate given the emerging, heterogeneous, and interdisciplinary nature of AI-related research in DCD, which encompasses diverse study designs, AI methodologies, data modalities, populations, and clinical purposes [29,30]. Rather than assessing intervention effectiveness or comparative efficacy, this review aimed to characterize how AI has been applied, in what contexts, and with what methodological approaches, as well as to identify research gaps and future directions. In this review, the term “intervention” refers to the application contexts of AI-supported tools rather than to the evaluation of their comparative clinical effectiveness. Consequently, no formal risk-of-bias assessment or meta-analysis was undertaken, consistent with scoping review methodology [31]. For the final chosen studies that were examined, this review aims to provide answers to the following research issues.

RQ1. How has artificial intelligence been used in the evaluation and intervention of DCD?

RQ2. What types of artificial intelligence methods (e.g., machine learning, deep learning, computer vision) and data modalities (e.g., video, wearable sensors, EEG, electronic health records) are used in AI-related DCD research?

RQ3. What study designs and levels of methodological maturity characterize the current evidence base on AI applications in DCD?

RQ4. What outcomes, key findings, and limitations are reported for AI-based approaches in the evaluation and intervention of DCD?

### 2.2. Inclusion and Exclusion Criteria

Studies were considered eligible for inclusion in this scoping review if they involved children or adolescents with DCD, including those with a formal clinical diagnosis, those classified as probable or at risk for DCD, or children presenting with motor impairments explicitly relevant to DCD as identified using standardized motor assessments. The review focused on empirical research that applied AI or AI-assisted approaches, defined as data-driven systems capable of inference, prediction, classification, or adaptive decision making. Eligible studies included those employing machine learning, deep learning, computer vision, or related AI methodologies, whether used as the primary analytical approach or as an integrated component supporting clinical evaluation or intervention.

Included studies were required to address at least one stage of the DCD care pathway, such as screening or early risk identification, assessment or evaluation, monitoring or outcome measurement, or intervention and rehabilitation. A wide range of empirical study designs was considered eligible, including randomized controlled trials, observational studies, AI development and validation studies, feasibility or pilot studies, and population-based prediction studies. Only peer-reviewed articles published in English were included.

Studies were excluded if they did not employ AI-based methods, relying instead on conventional statistical analyses, rule-based systems without learning or inference, or non-AI digital technologies such as augmented reality or robotics without an AI component. Research focusing on conditions other than DCD, including broadly defined dyspraxia without explicit alignment to DCD criteria, other neurodevelopmental disorders without DCD-specific analyses, or adult-only populations, was also excluded. In addition, non-empirical publications such as narrative or systematic reviews, theoretical or conceptual papers, opinion pieces, and co-design or technology development studies without AI-based evaluation or intervention outcomes were not considered eligible. Finally, studies lacking sufficient methodological transparency, including those that did not clearly report participant characteristics or AI methodology, were excluded (Table 1).

### 2.3. Database Screening and Selection Process

A systematic and comprehensive literature search was conducted to identify relevant studies examining the use of AI in the evaluation and intervention of DCD. The database selection process was designed to ensure broad coverage of interdisciplinary research spanning healthcare, rehabilitation, neuroscience, and computer science. The following electronic databases were searched, Web of Science, Scopus, PubMed, and IEEX, supplemented by Google Scholar to capture additional peer-reviewed and open-access publications that might not be indexed in the primary databases. The search strategy and study selection process were conducted in accordance with the PRISMA 2020 guidelines and the PRISMA extension for Scoping Reviews (PRISMA-ScR) [32]. In addition, a snowballing approach was applied by manually screening the reference lists of included articles to identify further relevant studies [33].

Search terms were developed iteratively to balance sensitivity and specificity and were adapted to each database using controlled vocabulary and Boolean operators. Keywords related to artificial intelligence and developmental coordination disorder were combined with terms referring to assessment, screening, monitoring, and intervention. Central search strings were defined a priori and are presented in Table 1. Advanced search filters and Boolean operators were applied within each database to refine the results. More specifically, in Scopus, the search was conducted using the TITLE-ABS-KEY field, combining terms related to DCD (e.g., “developmental coordination disorder,” DCD, dyspraxia), artificial intelligence and machine learning techniques (e.g., “machine learning,” “deep learning,” “neural network*,” “support vector machine*,” “random forest*,” “computer vision”), and clinical or motor-related applications (e.g., diagnosis, assessment, screening, classification, prediction, movement analysis, kinematics, gait). This search yielded 133 records. In PubMed, a combination of Medical Subject Headings (MeSH) terms and free-text keywords was used to maximize sensitivity. Terms related to DCD were combined with MeSH and title/abstract terms describing AI methodologies (e.g., “Machine Learning,” “Artificial Intelligence,” “deep learning,” “neural network*,” “support vector machine*,” “computer vision”) and outcome-related concepts such as diagnosis, assessment, screening, classification, prediction, movement analysis, kinematics, and gait. The PubMed search yielded 31 records. The Web of Science search employed topic-based fields (TS) and combined DCD-related terms with AI methodologies and motor assessment concepts, while also restricting results to pediatric populations using child- and adolescent-related terms. This search returned 16 records. To ensure coverage of AI and computational engineering research that may not be indexed in biomedical databases, IEEE Xplore was also searched using comparable combinations of DCD-related and AI-related terms. The IEEE Xplore search yielded 25 records (Table 2).

Across all databases, search strategies were adapted to the specific syntax and indexing systems of each platform, while maintaining conceptual consistency. No date restrictions were applied, and searches were limited to articles published in English. The reference lists of all included studies were also screened using a snowballing approach to identify additional relevant publications not captured through the database searches. All retrieved records were imported into reference management software for duplicate removal prior to screening.

Following the initial search, all retrieved records were imported into that Desktop (version 1.19.8; Elsevier, Amsterdam, the Netherlands), where duplicate references were identified and removed. The remaining records underwent a two-stage screening process. In the first stage, titles and abstracts were screened to assess relevance based on the predefined eligibility criteria. Priority was given to studies that included the search terms in both the title and abstract. Articles that clearly did not meet the inclusion criteria were excluded at this stage.

In the second stage, the full texts of potentially eligible studies were retrieved and examined in detail. During full-text screening, additional factors such as study design, population characteristics, AI methodology, and clinical relevance were considered to determine final eligibility. The study selection process was conducted collaboratively by one main reviewer and two additional independent reviewers. Initial screening and data organization were led by the main author, while two other researchers independently reviewed all eligible studies. Each reviewer classified studies as included, excluded, or uncertain. In cases of uncertainty or disagreement, discrepancies were resolved through discussion and consensus. When necessary, input from additional collaborators was sought to ensure methodological rigor and transparency. Following final study selection, the included studies were subjected to data charting and qualitative synthesis. Relevant information was extracted and organized into structured tables, and the findings were synthesized narratively. This process enabled a critical overview and evaluation of the existing evidence on AI applications in DCD, consistent with the objectives of a scoping review.

### 2.4. Data Extraction

Data from the selected studies included participant demographics, information (authors, year, country), specific information related to the research questions formulated, and aspects of the studies’ structure (study design, aim, conditions, variables/measure, major findings). More specifically, the data extraction process focused on systematically charting key characteristics of each study, including bibliographic details, study design, population characteristics, diagnostic status of DCD, and geographic context. In addition, detailed information related to AI applications was extracted, such as the clinical purpose of AI use (e.g., screening, assessment, monitoring, or intervention), the type of AI methods employed (e.g., machine learning, deep learning, computer vision), and the data modalities utilized (e.g., video, wearable sensors, EEG, electronic health records).

Further data items included the motor domains targeted (e.g., fine motor skills, gross motor coordination, gait, physical activity), study design and methodological maturity (e.g., AI development, feasibility, observational cohort, randomized controlled trial), and key outcomes and findings reported by the authors. Reported limitations of each study were also extracted to support identification of methodological gaps and areas for future research. Data extraction was initially performed by the main reviewer, who independently charted all included studies using the predefined framework. To enhance reliability and reduce potential bias, the extracted data were subsequently reviewed and verified by two additional researchers. Any discrepancies or uncertainties identified during this process were resolved through discussion and consensus. When clarification was required, the full-text articles were re-examined to ensure accurate interpretation of study details.

The extracted data were organized into structured tables to facilitate comparison across studies and to support a descriptive and narrative synthesis. Given the heterogeneity of study designs, AI methodologies, populations, and outcomes, quantitative synthesis was not pursued. Instead, the data charting process enabled a comprehensive mapping of the existing evidence, aligning with the objectives of this scoping review to characterize current applications of AI in DCD and to identify gaps in the literature.

### 2.5. Included Studies

As previously mentioned, the primary databases searched included Scopus, PubMed, and Web of Science. In addition, IEEE Xplore (IEEX) was searched to capture engineering- and technology-oriented studies relevant to AI-based methodologies. Google Scholar was used as a complementary source to identify additional peer-reviewed and open-access publications and to support citation tracking through snowballing. A total of n = 205 articles were located and screened for the final selection of the included articles after processing, inclusion, and representative criteria for the main part (n = 7) in the final selection for further research and analysis. The period of the literature review was January 2010–2025 [34].

**Figure 1:**
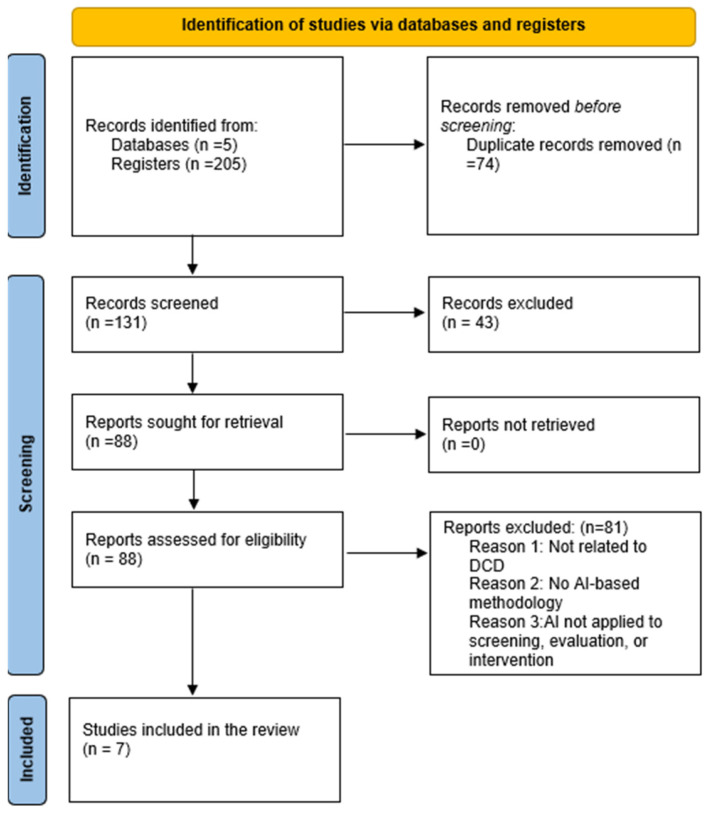
Prisma 2020 chart flow.

The first step in the selection process was the establishment of eligibility criteria. In addition, a list of keywords was developed to initiate the database search, using a range of search filters and Boolean operators to maximize the number of results. The selection procedure was carried out utilizing title and abstract screening in accordance with the eligibility criteria (n = 131) after duplicate studies were eliminated (n = 74). The next step in the process was the full-text screening after (n = 43) publications were excluded. The remaining studies (n = 88) underwent a comprehensive processing. In the final selection process, two independent reviewers debated whether the eligibility requirements applied after reviewing the full texts of (n =88) papers. The remaining (n =81) were removed since they did not fit the eligibility conditions. Consequently, n = 7 items were included in the final selection (Figure 1) [32].

### 2.6. Characteristics of Studies

A total of seven studies met the inclusion criteria and were included in this scoping review. The included studies were published between 2021 and 2026, reflecting the recent and emerging nature of AI applications in the evaluation and intervention of DCD [35,36,37,38,39,40,41]. Considerable heterogeneity was observed across studies in terms of study design, population characteristics, AI methodologies, data modalities, and clinical objectives.

**Figure 2:**
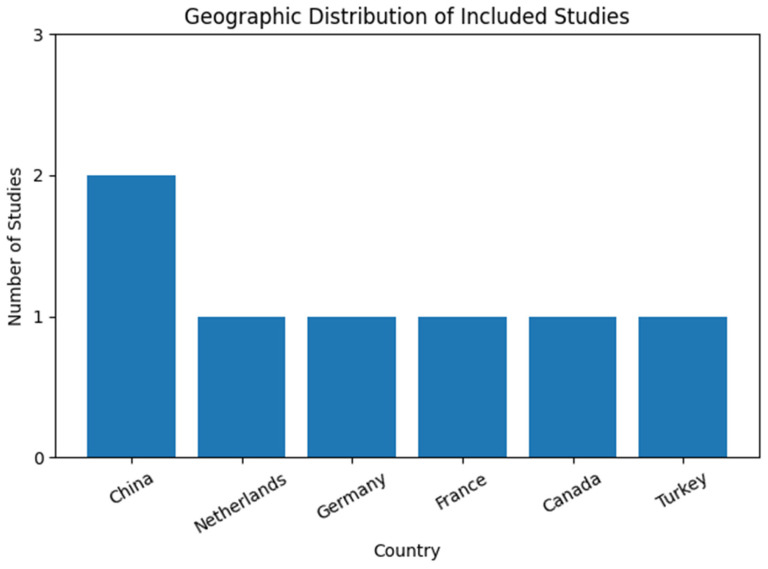
Country distribution of included studies.

The included studies were conducted across multiple geographic regions, primarily in high-income countries. European countries were most frequently represented, including the Netherlands, France, and Germany [35,36,40,41]. Additional studies were conducted in Turkey [39]; North America, namely Canada [37]; and East Asia, specifically China [38]. No studies from low- or lower middle-income countries were identified, indicating a geographic concentration of AI-related DCD research in economically developed regions (Figure 2).

**Figure 3:**
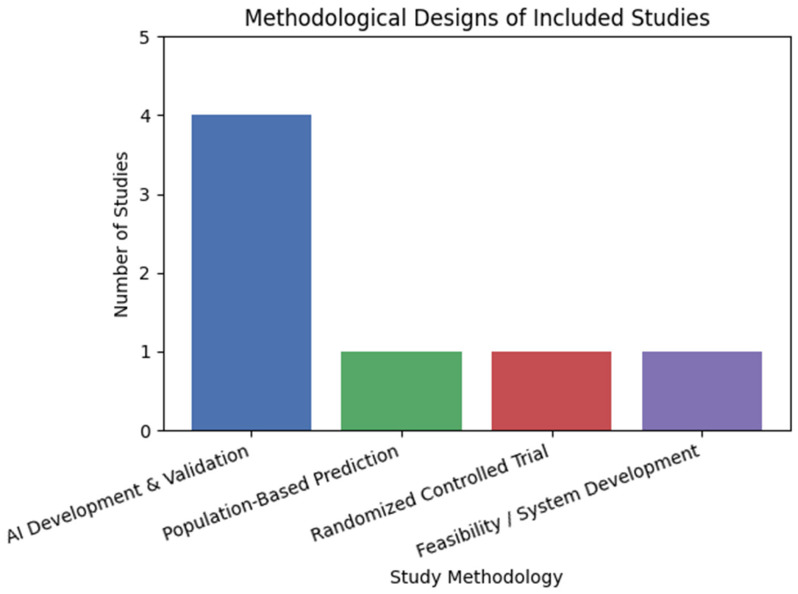
Methodology and study design of included studies.

The methodological approaches of the included studies varied substantially. Most studies employed observational designs or AI development and validation frameworks, focusing on the feasibility, classification accuracy, or predictive performance of AI models [35,36,41]. One study adopted a large-scale population-based prediction design, including both internal and external validation cohorts, to support early screening for DCD risk [38]. Only one randomized controlled trial was identified, evaluating the effectiveness of an AI-assisted occupational therapy intervention targeting handwriting skills in children at risk for DCD [39]. In addition, one study employed a feasibility and system development design to assess an AI-supported rehabilitation monitoring system during upper-limb therapy [40]. Overall, the evidence base is dominated by early-phase and exploratory research, with limited use of controlled experimental designs (Figure 3).

All included studies focused on pediatric populations, with ages ranging from preschool (5 years) to early adolescence (up to 12 years) [36,37,38,39]. Diagnostic certainty varied across studies. A minority included children with clinically diagnosed DCD based on standardized diagnostic criteria [41], whereas others focused on children classified as probable or at risk for DCD using screening tools or motor performance thresholds [37,39]. Two studies examined motor impairments relevant to DCD without confirming a formal diagnosis, positioning AI primarily as a screening or assessment-support tool rather than a diagnostic replacement [35,36]. Sex distribution was inconsistently reported across studies. When reported, samples were predominantly male, consistent with the higher prevalence of DCD in boys [36,37]. No study examined sex-specific differences in AI model performance or outcomes.

The primary clinical purpose of AI across the included studies was evaluation and assessment. Three studies focused on automated motor assessment, classification, or screening using AI models trained on kinematic, neurophysiological, or behavioral data [35,36,41]. Early screening and risk prediction were addressed in preschool populations using large-scale health data [37,38]. Ref. [40] has explored AI for monitoring during rehabilitation, providing real-time or post hoc analysis of movement during therapy sessions. Only one study evaluated an AI-assisted intervention, examining the effects of adaptive, technology-supported occupational therapy on handwriting performance [39].

A broad range of AI methodologies was reported, including traditional machine learning algorithms such as random forests, decision trees, support vector machines, and logistic regression [35,36,37,38], as well as deep learning and computer vision approaches applied to video-based gait and movement analysis [41] and sensor-based rehabilitation monitoring [40]. Data modalities varied widely and included markerless video recordings, wearable inertial sensors, electroencephalography (EEG), sensor-augmented toys, and electronic health records. Most studies relied on single-modality data, with limited exploration of multimodal AI approaches.

Reported outcomes primarily focused on technical performance metrics, such as classification accuracy, F1-score, and area under the curve, as well as feasibility and proof-of-concept outcomes [35,36,41]. Clinical effectiveness outcomes, long-term follow-up, and real-world implementation metrics were rarely reported. Most studies explicitly acknowledged methodological limitations, including small sample sizes, heterogeneous populations, and limited external validation [39,40,41].

## 3. Results

### 3.1. RQ1. How Has AI Been Used in the Evaluation and Intervention of DCD?

The most prevalent application of AI identified in this review relates to the evaluation and assessment of motor performance associated with DCD. Two studies employed machine learning and deep learning approaches to analyze motor behavior, movement patterns, or neurophysiological signals in order to support automated assessment or classification. Ref. [35] applied supervised machine learning models to EEG data to distinguish children with motor impairment consistent with DCD from typically developing peers, demonstrating the feasibility of AI-based neurophysiological classification. Similarly, ref. [36] used machine learning algorithms applied to data collected from sensor-augmented toys to predict fine motor impairment based on standardized motor assessment outcomes, positioning AI as a screening and assessment-support tool. Computer vision-based approaches were also used to automate the assessment of gross motor coordination. Ref. [41] developed and validated an AI system using markerless 2D gait video analysis to differentiate children with clinically diagnosed DCD from healthy controls and children with early-onset ataxia, highlighting the potential of video-based AI to provide objective gait assessment in clinical settings. In addition to task-specific motor assessment, AI was used to derive novel outcome measures from real-world activity data. Ref. [37] applied machine learning techniques to wearable accelerometer data to classify physical activity types in preschool children, revealing differences in walking and running behavior among children with probable or at-risk DCD that were not detectable using traditional intensity-based metrics. Three studies employed AI to support screening and early identification of DCD risk, particularly in younger populations. Ref. [38] developed a large-scale, population-based prediction model using electronic health record data to identify children at increased risk for DCD in early childhood. This approach explicitly framed AI as a pre-screening mechanism, enabling early identification and prioritization for further assessment rather than providing a definitive diagnosis. Similarly, studies focusing on motor impairment classification using standardized assessment thresholds (e.g., MABC-2 cut-offs) positioned AI as a tool to flag children who may require further diagnostic evaluation, rather than to confirm DCD diagnosis directly [35,36]. Ref. [40] explored AI applications for monitoring motor performance during rehabilitation by developing an AI-based system capable of recognizing and classifying upper-limb movements during therapy using wearable sensors and deep learning models. While the study focused primarily on technical feasibility and classification accuracy, it demonstrated the potential of AI to support objective monitoring and feedback during motor rehabilitation sessions. Ref. [39] conducted a randomized controlled trial examining the effects of an AI-supported occupational therapy intervention targeting handwriting performance in children at risk for DCD. The intervention incorporated adaptive feedback informed by AI algorithms and resulted in significant improvements in handwriting outcomes compared with a control condition. This study represents the highest level of evidence identified in this review and illustrates the potential for AI to contribute not only to assessment but also to therapeutic intervention.

The findings indicate that AI has been used in DCD literature predominantly to enhance evaluation, assessment, and screening, with comparatively limited application in intervention. AI systems were consistently framed as supportive technologies designed to augment existing clinical practices by improving objectivity, sensitivity, and scalability. Evidence supporting AI-assisted intervention remains scarce, underscoring the early developmental stage of this research field and the need for further controlled and longitudinal studies (Table 3).

### 3.2. RQ2. What Types of AI Methods and Data Modalities Were Used in AI-Related DCD Research?

Across the included studies, traditional machine learning algorithms were the most frequently used AI methods. Random forest models were commonly applied in both screening and assessment contexts, particularly when handling structured or semi-structured data such as accelerometer outputs or electronic health records [35,37,38]. Support vector machines, decision trees, and logistic regression models were also employed, especially in studies focusing on classification or prediction tasks related to motor impairment or DCD risk [36,38]. Other studies implemented deep learning approaches, particularly when working with high-dimensional or time-series data. Ref. [40] applied convolutional neural networks (CNNs), long short-term memory (LSTM) networks, and transformer architectures to wearable sensor data in order to recognize and classify upper-limb movements during rehabilitation tasks. Similarly, ref. [41] employed deep learning and ensemble learning techniques, including convolutional neural networks combined with gradient-boosted decision trees, to analyze gait patterns extracted from markerless video recordings. Overall, AI models were primarily developed and evaluated using supervised learning paradigms, with limited exploration of unsupervised or reinforcement learning approaches. Model explainability was addressed in only a small number of studies, most notably through the use of SHAP-based feature attribution in video-based gait analysis [41].

The data modalities used across the included studies were highly varied, reflecting different clinical and research objectives. Movement-based data constituted the most common modality, captured through multiple technological means. Markerless video-based motion analysis was used to assess gross motor coordination and gait patterns, enabling objective evaluation without the need for specialized motion capture systems [41]. Wearable sensor data, including inertial measurement units and accelerometers, were widely used to capture fine-grained movement characteristics during daily activities or rehabilitation tasks [37,41]. In addition, ref. [36] employed sensor-augmented toys, integrating embedded sensors to capture fine motor performance during play-based tasks. Neurophysiological data were less frequently used. Ref. [35] analyzed EEG signals to explore neural correlates of motor impairment consistent with DCD, applying machine learning models to classify motor-impaired and typically developing children. In contrast to movement-based data, EEG-based approaches remain relatively underrepresented in the current AI-DCD literature. One study diverged from performance-based data by using electronic health records (EHRs) to develop a population-level screening model for DCD risk [38]. This approach leveraged demographic, perinatal, and developmental variables rather than direct motor performance measures, highlighting the potential of AI for early identification using routinely collected data. Most included studies relied on single-modality data, focusing on either movement, neurophysiological, or health record information in isolation. Although studies combined multiple AI techniques, true multimodal data integration such as combining video, wearable sensors, and neurophysiological signals within a single AI framework was notably absent. This represents a methodological gap, given the multidimensional nature of DCD and its neurocognitive and motor underpinnings.

AI-related DCD research predominantly employs supervised machine learning models applied to movement-based data, particularly wearable sensors and video recordings. Deep learning approaches are emerging, especially in contexts involving complex temporal or visual data, while neurophysiological and multimodal AI applications remain limited. These findings suggest that current AI research in DCD prioritizes feasibility and interpretability but has yet to fully exploit the potential of multimodal and integrative AI frameworks.

### 3.3. RQ3. What Study Designs and Levels of Methodological Maturity Characterize the Current Evidence Base on AI Applications in DCD

The included studies demonstrate that the evidence base on AI applications in DCD is characterized primarily by early-phase methodological maturity, with most publications focused on model development, feasibility, and observational evaluation, and comparatively limited use of controlled experimental designs. Overall, the literature reflects a field that is progressing from proof-of-concept applications toward clinical translation, but remains largely in the development and validation stage rather than implementation or effectiveness evaluation.

Most included studies employed observational designs and/or AI development and validation frameworks. These studies were typically cross-sectional, focusing on training and evaluating supervised AI models to classify or predict motor-related outcomes relevant to DCD. For example, refs. [35,36] applied supervised machine learning models to neurophysiological and sensor-based motor data, respectively, using internal validation procedures such as cross-validation to assess classification performance. Similarly, ref. [41] used a cross-sectional dataset of clinically diagnosed children and controls to develop and validate markerless video-based gait classification models. One study used an observational cohort design with AI applied to enhance outcome measurement rather than to classify diagnostic status. Ref. [37] analyzed accelerometer data from a preschool cohort and used machine learning methods to derive physical activity types and quantify group-level behavioral differences, illustrating how AI can be used for refined measurement within epidemiological or developmental cohorts. A distinct methodological category was represented by [38], which implemented a population-based prediction model development design. This study demonstrated a higher level of maturity relative to most other included studies by incorporating both a very large development cohort and external validation across independent samples, thereby supporting more robust evaluation of generalizability and real-world screening potential. Finally, the interventional evidence base was limited. Only one study used a controlled experimental approach, with [39] conducting a randomized controlled trial evaluating an AI-assisted occupational therapy intervention for handwriting in children at risk for DCD. This represents the most methodologically rigorous evidence among included studies, but also highlights the scarcity of intervention trials in the field. In addition, ref. [40] employed a feasibility and system development design, focusing on technical validation of an AI-supported rehabilitation monitoring system rather than testing clinical effectiveness outcomes.

Across studies, AI applications were predominantly developed and evaluated using internal validation strategies, such as cross-validation or testing within a single dataset [35,36,41]. While these approaches support early feasibility and proof-of-concept evaluation, they provide limited evidence regarding external generalizability. External validation, which is often considered a key marker of methodological maturity and translational readiness, was explicitly incorporated in the population-level screening study by [38]. In contrast, most other studies did not report validation across independent sites or datasets, and few addressed implementation considerations such as clinical workflow integration, interpretability for clinicians, or prospective performance in real-world settings.

The overall maturity of the evidence base suggests that AI applications in DCD are currently concentrated in early translational phases, with emphasis on developing and testing models under controlled conditions. Relatively few studies have progressed to designs capable of evaluating whether AI improves clinical decision making, reduces assessment burden, enhances treatment planning, or yields sustained functional improvements. The limited number of intervention studies and the predominance of feasibility-oriented outcomes indicate that the field has not yet established a strong foundation of evidence supporting routine clinical adoption [39,40]. These findings highlight the need for future research to prioritize multisite validation, longitudinal designs, and controlled clinical trials to support translation of AI applications from proof-of-concept to clinical utility.

### 3.4. RQ4. What Outcomes, Key Findings, and Limitations Were Reported

Across the included studies, outcomes were reported primarily in two forms: technical performance metrics (e.g., accuracy, F1-score, AUC) for AI models supporting evaluation or screening, and clinical outcome measures for the small subset of intervention-focused work. Overall, the evidence base indicates promising feasibility and performance of AI methods in detecting motor-related patterns relevant to DCD; however, it is also characterized by recurring methodological limitations, including small or heterogeneous samples, variable diagnostic certainty, limited external validation, and restricted clinical translation.

Other studies reported model performance in terms of classification or prediction accuracy, demonstrating that AI can identify motor-related patterns associated with DCD or DCD-relevant impairment under specific conditions. Ref. [35] reported very high classification performance using a machine learning model trained on EEG spectral features to distinguish children with motor impairment consistent with DCD from controls. Ref. [36] similarly demonstrated that machine learning models trained on sensor-augmented toy data were able to predict fine motor impairment (defined using standardized assessment outcomes), supporting the feasibility of play-based, sensor-driven assessment support. In video-based gross motor assessment, ref. [41] reported moderate-to-good overall classification performance for a markerless gait analysis pipeline distinguishing children with DCD, early-onset ataxia, and healthy controls. Importantly, while overall performance was acceptable, the study noted that DCD classification was less robust than classification of the other groups, suggesting that gait signatures in DCD may be more heterogeneous and less distinct than in other movement disorders. Outcome measurement studies suggested that AI can enhance sensitivity to meaningful behavioral differences. Ref. [37] reported that machine learning-derived physical activity classifications from accelerometer data detected group differences in walking and running among preschool children with probable or at-risk DCD that were not captured using conventional physical activity intensity metrics. This supports the added value of AI for generating more granular, behaviorally relevant outcomes in real-world contexts. For early screening and risk prediction, ref. [38] reported moderate predictive performance for identifying DCD risk in early childhood using electronic health record data, highlighting the feasibility of population-level pre-screening and early identification. This study also showed reduced predictive performance when applied to older children, suggesting that risk-factor-based models may be most useful in preschool screening contexts.

Ref. [40] reported outcomes related to rehabilitation and intervention, showing a high performance of deep learning models for recognizing and classifying upper-limb movements during therapy tasks using wearable sensors, and demonstrating technical feasibility for AI-supported monitoring. However, clinical outcomes were not evaluated and DCD-specific results were not reported separately from other neurodevelopmental conditions included in the sample. Only one study reported direct clinical effectiveness outcomes. Ref. [39] presented significant improvements in handwriting outcomes following an AI-supported occupational therapy intervention for children at risk for DCD. This study provides preliminary evidence that AI-assisted therapeutic systems can contribute to clinically meaningful improvements, although broader conclusions are limited by sample size and population definition.

Despite promising findings, limitations were consistently reported across the included studies. A recurring limitation was sample size and representativeness, particularly in studies using complex sensor, EEG, or video-based pipelines, which often relied on relatively small datasets or single-site samples [35,36,41]. In addition, heterogeneity in diagnostic certainty was common with studies focusing on at-risk, probable, or motor-impaired groups rather than clinically diagnosed DCD, limiting diagnostic generalizability and interpretability in clinical contexts [36,37,39]. Another frequent limitation involved validation and generalizability. Many studies relied on internal validation strategies (e.g., cross-validation) without independent external datasets, raising concerns regarding model robustness and transferability to other settings [35,36,40]. Ref. [38] represented an exception by incorporating external validation, but also demonstrated that predictive performance may drop outside the developmental period targeted by the model, underscoring the challenge of generalizing AI models across age groups. The included studies also highlighted challenges in clinical translation. Rehabilitation monitoring work emphasized technical classification accuracy but did not assess whether AI monitoring improves functional outcomes or clinical decision making [40]. Similarly, assessment-focused studies reported model performance but did not evaluate feasibility in routine clinical workflows or consider implementation factors such as clinician interpretability, usability, or longitudinal stability of measurements [36,41].

## 4. Discussion

A central insight emerging from this scoping review is that DCD remains systematically under-recognized, not only in clinical and educational systems [42,43] but also within the rapidly expanding domain of AI in healthcare. Despite its high prevalence, persistence across the lifespan, and substantial impact on participation and quality of life, DCD continues to receive less scientific and technological attention than other neurodevelopmental conditions, particularly autism spectrum disorder and attention-deficit/hyperactivity disorder [9,44].

While AI has been widely adopted to support assessment, diagnosis, and intervention in other neurodevelopmental disorders, this review shows that its use in DCD remains extremely limited. Existing applications are few, largely exploratory, and primarily focused on assessment and screening. This pattern suggests that DCD risks being marginalized not only in clinical practice but also within emerging digital health innovation, potentially reinforcing existing inequalities in research attention and resources allocation.

One explanation for this may lie in the conceptual complexity and heterogeneity of DCD. Unlike conditions characterized by relatively discrete behavioral and neurocognitive markers, DCD encompasses a wide range of motor learning difficulties that vary across tasks, contexts, and developmental stages. This variability poses challenges on both traditional research designs and data-driven AI models, which often depend on clearly defined labels and stable phenotypes [9,44,45,46].

Regarding the current use of AI on DCD, the findings of this review indicate that running AI applications in DCD overwhelmingly reflect a diagnostic-centric paradigm, emphasizing classification, screening, and objective measurement. These approaches are valuable, particularly given the limitations of existing tools, such as subjectivity, time burden, and limited sensitivity to subtle motor patterns. However, exclusive focus on evaluation risks reinforcing a narrow view of DCD as a condition to be identified rather than actively and effectively treated. This imbalance mirrors long-standing critiques within DCD research, where substantial effort has been devoted to describing deficits and neural correlates with comparatively less attention being given to intervention processes and long-term functional outcomes. If AI remains confined to assessment, it risks reproducing these limitations rather than advancing clinical practice.

Regarding clinical settings and practice, occupational therapists (OTs) [47,48] and physiotherapists (PTs) [49] play a central role in the assessment and management of DCD, particularly in pediatric populations. DCD is not addressed through a single standardized protocol but through individualized, task-oriented, and content-sensitive interventions grounded in motor learning theory and occupational performance models.

This review suggests that AI holds considerable, yet largely untapped, potential to support these therapeutic processes. Beyond automating assessment, AI systems could help identify individual motor learning profiles, track responses to intervention over time, support adaptive task grading, and optimize feedback timing, frequency and modality. For OTs and PTs, AI could also be integrated into occupation-based frameworks [50,51] to support goal setting, task analysis [52], and participation-focused outcomes. AI-driven analysis of movement variability, balance control, or gait adaptation could further inform individualized motor training strategies. Furthermore, such applications would need to be designed explicitly around therapeutic reasoning and clinical logic, rather than focusing entirely on pattern recognition.

A promising direction involves aligning AI systems with established motor learning principles, such as variability of practice, contextual interference, and feedback optimization. Individuals with DCD often show atypical motor learning trajectories [5], and AI systems capable of adapting task demands in real time could offer meaningful clinical benefits. However, none of the included studies explicitly grounded AI applications in motor learning theory [53] or rehabilitation science models [54]. This highlights a disconnect between technological development and therapeutic theory. Future AI systems should move beyond performance metrics to engage with how learning proceeds over time, particularly in developmental populations.

Another promising yet unexplored prospect is the use of chatbots and large language models (LLMs) [55,56]. Although none of the reviewed studies employed conversational AI, these tools may have value as clinical reasoning and knowledge translation supports. LLMs could assist therapists by synthesizing evidence across motor learning theories, intervention models, and professional frameworks. They could also support hypothesis generation during assessment, reflective practice, and documentation, offering structured decision-support prompts rather than prescriptive answers.

Moreover, an LLM could help an OT link observed motor difficulties to relevant theoretical constructs (such as internal modeling deficits or executive function demands) and suggest evidence-informed interventions aligned with specific frames of reference. Similarly, PTs could use such systems to explore movement analysis interpretations or progression strategies grounded in current research. Finally, these tools should not function as autonomous decision makers; rather, they should act as augmentative cognitive supports that enhance, rather than replace, professional expertise [56]. Ethical safeguards, transparency, and clinical oversight are essential to prevent misuse or over-reliance [57,58]. Despite providing a comprehensive mapping of AI applications in DCD, several limitations should be acknowledged. First, the available evidence base remains small and heterogeneous, with only a limited number of studies meeting the inclusion criteria, reflecting the early stage of AI research in this clinical population [35,36,37]. Most included studies focused on assessment or screening rather than intervention, and only one randomized controlled trial was identified, restricting conclusions about clinical effectiveness [39]. In addition, many studies relied on relatively small, single-site samples, which limits the generalizability of findings and the robustness of model validation [36,40]. Moreover, diagnostic certainty varied across studies, with some including children identified as “at risk” for DCD rather than clinically diagnosed cases, potentially introducing variability in the populations examined [5,38]. Also, AI methodologies were predominantly based on supervised machine learning using structured datasets, with limited exploration of multimodal, longitudinal, or real-world clinical data [37,41]. Finally, this scoping review did not assess risk of bias or intervention efficacy, in line with its methodological purpose, and was restricted to English-language, peer-reviewed publications, which may have excluded relevant studies from other regions or gray literature [32]. These limitations highlight the need for larger, multicenter, theory-informed, and clinically integrated research to support the safe and effective translation of AI tools into DCD assessment and intervention practice.

Apart from these limitations, the present scoping review is the first to provide a valuable overview of the current landscape of AI applications in DCD and highlights important directions for future research and clinical practice.

## 5. Conclusions

This is the first study that delves into investigating the broad use of AI in the DCD population. The study collected and analyzed the bibliography systematically, collecting data, highlighting the gap, and offering valuable insights about future research studies and methodology. A major challenge identified across the included studies was the limited attention being given to clinical implementation. Many AI applications demonstrated promising technical performance but fail to address usability, interpretability, or integration into everyday clinical workflows. For therapists working with DCD, time constraints, resource limitations, and contextual demands are critical considerations. Future research must therefore prioritize co-design approaches involving OTs and PTs, ensuring that AI systems are usable, interpretable, and aligned with real-world therapeutic contexts. Without this alignment, even technically sophisticated AI tools are unlikely to achieve meaningful clinical adoption. Based on the findings of this review, future research should prioritize AI-assisted intervention studies grounded in motor learning and rehabilitation theory; longitudinal and adaptive models that track individual change over time; multimodal AI systems integrating movement, cognitive, and contextual data; exploration of LLMs and conversational AI for clinical reasoning and education; and rigorous evaluation of clinical impact, usability, and ethical implications (Table 3).

## Figures and Tables

**Table 1:** Inclusion–exclusion criteria established.

Inclusion Criteria (IC)	Exclusion Criteria (EC)
Studies involving children adolescents or adults explicitly addressing DCD (DCD, probable DCD, or individuals identified as at risk for DCD) or motor coordination difficulties clearly aligned with DCD definitions based on standardized motor assessments	Studies focusing on other neurodevelopmental conditions (e.g., ASD, ADHD) without DCD-specific analysis
Empirical studies applying artificial intelligence or AI-assisted methods (e.g., machine learning, deep learning, computer vision)	Studies using only conventional statistical methods, rule-based systems, or non-AI digital technologies
AI applied to at least one stage of the DCD care pathway (screening, assessment, monitoring, intervention, or rehabilitation)	Studies using technology for non-clinical purposes (e.g., educational gaming, usability testing without clinical outcomes)
Quantitative or mixed-methods empirical study designs, including development, validation, feasibility studies, observational studies, or randomized controlled trials	Non-empirical publications, including reviews, editorials, opinion papers, theoretical or conceptual articles
Peer-reviewed journal articles published in English	Conference abstracts, protocols, gray literature, or articles without full text available
Studies reporting sufficient methodological detail, including participant characteristics and AI methodology	Studies with insufficient methodological transparency or unclear reporting of sample or AI approach

**Table 2:** Research data strings performed per database.

Database	Search String
Scopus	TITLE-ABS-KEY ((“developmental coordination disorder” OR DCD OR dyspraxia) AND (“machine learning” OR “deep learning” OR “neural network*” OR “support vector machine*” OR “random forest*” OR “computer vision”) AND (diagnos* OR assessment OR screening OR classification OR prediction OR “movement analysis” OR “motor assessment” OR kinematic* OR gait))
PubMed	(“Developmental Coordination Disorder”[MeSH] OR “developmental coordination disorder”[Title/Abstract] OR DCD[Title/Abstract] OR dyspraxia[Title/Abstract]) AND (“Artificial Intelligence”[MeSH] OR “Machine Learning”[MeSH] OR “deep learning”[Title/Abstract] OR “neural network*”[Title/Abstract] OR “support vector machine*”[Title/Abstract] OR “computer vision”[Title/Abstract]) AND (diagnos*[Title/Abstract] OR assessment[Title/Abstract] OR screening[Title/Abstract] OR classification[Title/Abstract] OR prediction[Title/Abstract] OR “movement analysis”[Title/Abstract] OR kinematic*[Title/Abstract] OR gait[Title/Abstract])
Web of Science	TS = (“developmental coordination disorder” OR DCD OR dyspraxia) AND TS = (“machine learning” OR “deep learning” OR “neural network*” OR “computer vision”) AND TS = (diagnosis OR assessment OR screening OR classification OR “movement analysis” OR kinematic* OR gait) AND TS = (child* OR pediatric* OR paediatric* OR adolescent*)
IEEE Xplore	(“developmental coordination disorder” OR DCD OR dyspraxia) AND (“artificial intelligence” OR “machine learning” OR “deep learning” OR “neural network*” OR “computer vision”) AND (assessment OR classification OR screening OR prediction OR “movement analysis” OR gait)

**Table 3:** Summary of the analysis of the included studies.

Study	Year	Country	Sample Size and Demographics	DCD Status	AI Purpose	AI Methods	Data Modality	Motor Domain	Specific Study Design	Key Results	Main Limitations
Demirci et al.	2025 [39]	Turkey	n = 42; 8–12 yrs; intervention (21), control (21)	At risk for DCD (DCDQ)	Intervention	ML-driven adaptive feedback	Digital handwriting platform	Handwriting/fine motor	Randomized controlled trial (parallel-group, assessor-blinded)	Significant improvements in all handwriting domains (legibility, speed, spacing, alignment) in the AI-assisted group compared with controls	At-risk rather than diagnosed DCD; small sample size; short intervention duration; no long-term follow-up
Marhraoui et al.	2025 [40]	France	n = 30; 7–10 yrs; 24 boys/6 girls	Mixed sample incl. DCD (subgroup NR)	Rehabilitation support and monitoring	CNN, LSTM, Transformer	Wearable IMUs	Upper-limb coordination	Feasibility study with AI system development	Transformer model achieved high accuracy (≈82–96%) for activity and hand-use recognition during therapy tasks	Small sample; mixed diagnoses; no isolated DCD outcomes; no clinical effectiveness evaluation
Buettner et al.	2021 [35]	Germany (dataset: CZ)	n = 28; 7–10 yrs; 12 motor-impaired, 16 controls	Motor-impaired DCD (MABC-2)	Diagnosis/screening	Random Forest ML	EEG	Neural motor correlates	Secondary data analysis with supervised ML	Very high classification accuracy (>99%) distinguishing motor-impaired children from controls	Small sample; DCD defined by motor impairment only; no external validation; limited clinical interpretability of EEG features
Letts et al.	2025 [37]	Canada	n = 497; 4–5 yrs; 56.5% boys	Probable DCD (61); at risk (115)	Outcome measurement	Random Forest ML	Accelerometers	Physical activity behavior	Observational cohort with ML-based classification	ML-derived metrics revealed reduced walking and running time in pDCD/DCDr groups not detected by traditional intensity measures	Not diagnostic; AI model not trained specifically on DCD; observational design; limited motor-task specificity
Dai et al.	2025 [38]	China	Dev: n = 150,948; Val: n = 1359	Possible + confirmed DCD	Early screening/risk prediction	Logistic regression; Random Forest	EHR data	Global motor risk	Population-based prediction with internal and external validation	Screening model achieved moderate discrimination (AUC ≈ 0.70) in preschool children, supporting early risk identification	Predictive (not diagnostic); reduced performance in older children; relies on indirect risk factors rather than motor performance
Tang et al.	2026 [41]	China	n = 83; 17 DCD, 32 EOA, 34 controls	Clinically diagnosed DCD	Automated assessment	CV + ML (XGBoost, SHAP)	Markerless 2D gait video	Gait/gross motor	Cross-sectional AI development and validation	Best model achieved F1 ≈ 0.73; EOA and controls classified well, DCD recall lower, highlighting gait heterogeneity	Small DCD subgroup; clip-based modeling inflates data points; limited sensitivity for DCD-specific gait patterns
Brons et al.	2021 [36]	The Netherlands	n = 95; mean age 7.8 yrs	Fine motor problems (≤16 th % MABC-2)	Assessment/screening	DT, KNN, LR, SVM	Sensor-augmented toy	Fine motor skills	Observational ML development study	ML models predicted fine motor impairment with good accuracy using toy-based sensor data	No formal DCD diagnosis; screening-level outcomes only; single-task assessment; no longitudinal validation

## Data Availability

No new data were created or analyzed in this study.
